# The Period of Infectivity of Venereal Disease

**Published:** 1922-01

**Authors:** 


### THE PERIOD OF INFECTIVITY OF VENEREAL DISEASE.

THE patient who has suffered from Gonorrhœa or Syphilis wishes, as soon as his immediate and distressing symptoms are alleviated, to know how soon he will be free of infection. Often enough the question is asked, when will the patient be fit to marry ? The answer is one which often has perplexed medical practitioners both in private and in public work. To give a man or woman a clean bill of health after venereal disease is a grave responsibility, and an error on the part of the physician may lead to the ruin of more than one life.

The report has recently reached us of a large Conference on Venereal Disease held in America at which every public and social interest was represented. The wide subject of Venereal Disease was discussed in detail and the Conference came to several important conclusions.

With regard to the eligibility for marriage of a person who has suffered from syphilis, this Conference was of the opinion that the following medical considerations apply :—

1. The eligibility for marriage of the person who has or has had syphilis depends in the main upon the possibility of his transmitting the disease.

2. The impossibility of absolutely determining by arbitrary rule the limits of infectivity in all cases has been admitted.

3. The problem may be more difficult of solution in women than in men, owing to the paucity of clinical and laboratory evidence of the disease in the former.

4. The clinical experience of many years has justified as reasonably safe, the following fundamental requirements :

(*a*) Three years of effective treatment.

(*b*) Two additional years of freedom from all signs and symptoms of the disease, under medical observation.

5. It is recognised that special types of cases may call for special interpretation, which, however, in all cases should be founded on the basic principles of effective treatment and prolonged painstaking observation for signs of recurrent or active syphilis.

6. In view of the inevitable element of uncertainty, however small, the prospective marital partner of a person who has or has had syphilis should be informed before marriage of the status of the case.

7. Medical examination to establish the presence or absence of syphilis before marriage should include not merely a blood Wassermann test, but an examination, clinical and serologic, of the entire body. If evidence of a previous or probable syphilitic infection presents, such examination should be especially searching, may include a period of observation, and should be interpreted by an expert.

The following extract gives the opinion of this all-American Conference regarding the period of infectivity and the cure of gonorrhœa :—

With our present knowledge it is not scientifically and medically practicable to establish a standard for determining when gonorrhœa is cured. This statement, however, must not be understood as reflecting upon the ability of any specialist to make such a decision.

In the female the condition is complicated, on account of the fact that the primary manifestations may be so slight as to cause the patient no annoyance, but particularly on account of the tendency of the disease to become latent. This latency is due to the fact that a minimal number of gonococci may be retained for months in a quiescent state in the depths of the cervical glands, and under certain conditions suddenly begin to multiply and give rise to an extension of the disease, accompanied by clinical symptoms. Accordingly, the disappearance of the initial clinical symptoms and the apparent freedom of the cervical secretion from gonococci do not necessarily indicate that the disease is cured.

On the other hand, in the male it is practicable to establish a reasonable standard for determining when gonorrhœa has been " probably cured." The criteria are based upon two distinct entities—(*a*) cure of the infection ; (*b*) cure of the lesions of the disease.

In gonorrhœa, whether in the male or female, the duration of infectiousness has but a single slight relation to duration of the lesions—that is, the symptoms may continue long after the infectiousness has ceased, and, conversely, infectiousness may persist after all obvious symptoms have ceased.

As the problem is confined exclusively to the question of infectiousness, the word " cure," as here used, will be understood as equivalent to cessation of infectiousness.

There is no combination of clinical facts which constitutes conclusive evidence of a cure of gonorrhœa in the male. Therefore, the determination as to whether cure has been effected depends upon the acumen and skill of the physician. The patient may be assured that he is probably cured if all of the following criteria are fulfilled :

(*a*) No urethral discharge whatever for one month, during which time the patient is under observation without treatment.

(*b*) The first ounce of urine passed in the morning free from any cloud of pus, as examined in a glass or bottle by a strong light.

(*c*) All shreds in the urine continue to float at least two minutes after agitation of the fluid has ceased.

(*d*) Massage of the prostate and seminal vesicles expresses a fluid which, when examined under the microscope, is found to contain no gonococci or pus cells.

(*e*) It should be possible to pass a 26-F sound into the bladder, and examination the following day should satisfy conditions *a* and *b*.

(*f*) If these findings are confirmed at a second examination one month later, during which time there have been no symptoms or treatment, the patient should be regarded as cured.

All other cases should be examined by an expert, in order to determine whether or not they are cured.

